# Lactic Acid Bacteria and Bacteriocins: Novel Biotechnological Approach for Biopreservation of Meat and Meat Products

**DOI:** 10.3390/microorganisms10102058

**Published:** 2022-10-18

**Authors:** Dipanwita Bhattacharya, Pramod Kumar Nanda, Mirian Pateiro, José M. Lorenzo, Pubali Dhar, Arun K. Das

**Affiliations:** 1 Department of Livestock Products Technology, Faculty of Veterinary and Animal Sciences, Banaras Hindu University, Varanasi 221005, India; 2 Eastern Regional Station, ICAR-Indian Veterinary Research Institute, 37 Belgachia Road, Kolkata 700037, India; 3 Centro Tecnológico de la Carne de Galicia, Avd. Galicia n° 4, Parque Tecnológico de Galicia, San Cibrao das Viñas, 32900 Ourense, Spain; 4 Área de Tecnoloxía dos Alimentos, Facultade de Ciencias, Universidade de Vigo, 32004 Ourense, Spain; 5 Laboratory of Food Science and Technology, Food and Nutrition Division, University of Calcutta, 20B, Judges Court Road, Alipore, Kolkata 700027, India

**Keywords:** novel bacteriocins, lactic acid bacteria, natural antimicrobials, quality and safety, meat and meat products

## Abstract

Meat and meat products are perishable in nature, and easily susceptible to microbial contamination and chemical deterioration. This not only results in an increased risk to health of consumers, but also causes economic loss to the meat industry. Some microorganisms of the lactic acid bacteria (LAB) group and their ribosomal-synthesized antimicrobial peptides—especially bacteriocins—can be used as a natural preservative, and an alternative to chemical preservatives in meat industry. Purified or partially purified bacteriocins can be used as a food additive or incorporated in active packaging, while bacteriocin-producing cells could be added as starter or protective cultures for fermented meats. Large-scale applications of bacteriocins are limited, however, mainly due to the narrow antimicrobial spectrum and varying stability in different food matrixes. To overcome these limitations, bioengineering and biotechnological techniques are being employed to combine two or more classes of bacteriocins and develop novel bacteriocins with high efficacy. These approaches, in combination with hurdle concepts (active packaging), provide adequate safety by reducing the pathogenicity of spoilage microorganisms, improving sensory characteristics (e.g., desirable flavor, texture, aroma) and enhancing the shelf life of meat-based products. In this review, the biosynthesis of different classes of LAB bacteriocins, their mechanism of action and their role in the preservation of meats and meat products are reviewed.

## 1. Introduction

Meat and meat-based food products are an excellent source of good-quality protein, and contain lipids and micronutrients, such as vitamins and minerals, that are required for the human diet [1,2]. However, meat and its products are perishable in nature, as they offer a favorable environment for microbial growth and activity of a wide spectrum of spoilage organisms. The presence of spoilage microorganisms, including several strains of *Enterobacteriaceae*, *Staphylococcus* spp., *Micrococcus* spp., *Brochothrix* spp., *Pseudomonas* spp. and lactic acid bacteria, play a vital role in the deterioration process of meat and meat products [3]. Various intrinsic factors such as pH (5.5–6.0), high water activity (0.99), readily available nutrient contents and extrinsic factors such as temperature and oxygen concentration make the environment conducive for their growth [4,5]. The deterioration process brings about undesirable changes in the quality, and alters color, texture and flavour, making the meat unsuitable for human consumption [6,7]. Meat contaminated with pathogenic organisms like *E. coli*, *Shigella*, *Bacillus* spp., *Clostridium* spp. and *Listeria* spp. can also pose a risk to human health [8,9]. Furthermore, the consumption of ready-to-eat meat products contaminated with several pathogenic microorganisms such as *Salmonella* spp., *Listeria monocytogenes* and *Escherichia coli* are often linked to several foodborne illness outbreaks [10]. Out of various species of the genus *Listeria* viz., *L. monocytogenes*, *L. ivanovii* and *L. seeligeri*, *L. monocytogenes* is a persistent organism, and can contaminate meat or meat products in any adverse conditions [11]. Furthermore, *Listeria* shows higher pathogenicity as compared to several non-spore-forming bacteria. The organism is also reported to exhibit moderate resistance, mainly to traditional food preservation techniques, and therefore may require additional actions to avert their prevalence in foods [11].

Microbial contamination is therefore a challenging and significant issue, as it brings in serious quality issues and safety aspects in the meat industry [12]. As the purpose of the meat industry is to produce safe food products with a longer storage life, several conventional methods like drying, freezing, packaging, canning, curing or dehydration [13,14], as well as chemical treatment methods, are employed to inhibit and/or inactivate pathogenic microorganisms [15,16]. At the industrial level, thermal processing and chemical preservatives are also widely used techniques for meat preservation. However, loss of nutritional quality, loss of taste and the production of undesirable carcinogenic substances such as nitrosamine have limited their uses in the commercial market.

During the last few years, the demand for quality foods or food products that have high nutritional value, but are synthetic chemical-free, has increased due to the changing lifestyle and eating habits of consumers. As the consumption of fresh meat and meat-based products is increasing day by day, due to the shift in the dietary habits of consumers towards abundant protein intake, quality and safety issues in meat production and preservation are a matter of concern [6]. Adequate precautionary measures are therefore needed to reduce the risk for microbial attack, starting from the slaughtering process to the packaging and storage of products.

To overcome these challenges, the food manufacturing industry is in search of novel natural alternatives that act as preservatives, provide adequate microbiological safety and offer a better shelf life to products. Many studies have revealed that few microorganisms from the lactic acid bacteria (LAB) groups viz. *Lactococcus*, *Lactobacillus*, *Pediococcus*, *Leuconostoc*, *Streptococcus*, *Enterococcus*, etc. could be added/used as starter/bioprotective cultures in meat-based products [17]. These microbial groups are able to inhibit the growth and other activities of meat spoilage bacteria, and offer better opportunities as natural and efficient food preservatives, as well as a good alternative to chemical compounds [18,19]. LAB metabolites, especially bacteriocins (e.g., nisin, enterocin, plantaricin, pediocin, pentocin, pneumocyclicin, sakacin) are of great interest to the dairy, meat and other food industries. Bacteriocins are small antimicrobial peptides that are quite effective against food pathogens such as *E. coli* O157:H7 and *Salmonella* sp., apart from spoilage microorganisms. Furthermore, bacteriocins are heat stable, and are considered safe for consumption, having fewer effects on human microbiota [18,20]. In recent days, a number of novel bacteriocins have been isolated from different kinds of dairy and fermented meat products [21,22]. For example, a simple crude curd extract from Indian curd (a fermented dairy product) could promisingly be used as food biopreservative due to its heat stability and strong antimicrobial action on various pathogenic microorganisms such as *Bacillus cereus* and *Salmonella* Typhimurium [23]. In this review, we will shed light in a concise way about the biosynthesis of different classes of bacteriocins from LAB, their mechanism of action and their role in meat and meat product preservation.

## 2. Lactic Acid Bacteria as Starter and Not Starter Culture

The LAB group includes more than 25 *Lactobacillus* genera, including *Lactobacillus*, *Acetilactibacillus*, *Agrilactobacillus*, *Amylolactibacillus*, *Furfurilactobacillus*, *Fructilactobacillus*, *Holzapfelia*, *Latilactobacillus*, *Lactiplantibacillus*, *Loigolactibacillus*, *Paralactobacillus*, *Schleiferilactobacillus*, etc. [24]. Besides *Lactobacillus*, *Streptococcus*, *Carnobacterium*, *Enterococcus*, *Lactococcus*, *Pediococcus* and *Weissella* are also under in the LAB group [22,25,26].

Several researchers have evaluated the use of single or multiple LAB strains in meat and meat products. These strains with favorable salt concentration, pH, temperature, lactose or dextrose are also reported to give desirable results by not only reducing pathogen counts, but also improving the shelf life of meat products by controlling lipid oxidation. Sakaridis et al. [27] evaluated the efficacy of *Ligilactobacillus salivarius* (10^6^ cfu/g) to inhibit the growth of *Salmonella* spp. and *L. monocytogenes* (both at 10^4^ cfu/g) in chicken skin stored at 7 °C for 6 days. The researchers observed a reduction of 0.5 log cfu/g in *Salmonella* spp. and 0.7 log cfu/g in *L. monocytogenes* during the storage period of the sample. Except for the reduction of pH value, no adverse sensory characteristics were noticed.

The use of some LAB strains like Lactobacillus in meat have been reported to exert antifungal activity against *Aspergillus fumigatus* and *A. niger* [28,29]. Likewise, bacteriocin or non-bacteriocin producing LAB strains are also reported as good fermentative cultures for sausages. Gelinski et al. [30] reported a 2.4 log cycle reduction of *Salmonella enterica* serotype Choleraesuis after 7 days of storage at 10 °C in fresh pork sausages inoculated with Lactobacillus sakei. Moreover, Kamiloğlu et al. [31] studied the positive effect of *Lactiplantibacillus plantarum* against *L. monocytogenes* in sucuk, a unique Turkish dry fermented sausage. The researchers observed a reduction in *L. monocytogenes* counts ranging from 1 to 2.7 log units with the different *L. plantarum* strains. Such inhibition was due to acidification during ripening or the release of antimicrobial metabolites. Nikodinoska et al. [32] also found a similar result in ‘Chorizo’ pork-based sausage, where *L. plantarum* showed pathogenicity against *L. monocytogenes*. In another study, Zanette et al. [33] recorded a reduction of 1.7 log cfu/g in *L. monocytogenes*, when *L. plantarum* was used as a starter culture in pork colonial sausages.

Although live LAB strains can show maximum antimicrobial properties, improve gut health and bring some desirable sensory changes; improper handling or strain selection may exert some adverse effects, such as slime formation, off-odors, or unwanted textural and color changes in meat, which may cause health hazards [34].

## 3. Bacteriocin

Bacteriocins are active metabolic peptides that are ribosomally synthesized by certain LABs or non-LABs. These are characterized by being non-toxic, and are either electrically neutral or positively charged. Bacteriocins produced by different LABs differ from each other by their unique biochemical, structural, genetic, ecological and metabolic activity. Biopreservation by bacteriocins is a promising area, due to their high specificity towards multi-drug resistant pathogens, which means they may be used as an alternative to antibiotics. Being heat stable, colorless, odorless, stable in a wide pH range, and able to be inactivated by proteolytic enzymes, these bacteriocins are used as biotechnological tool in the food and pharmaceutical industries. Pediocin from *Pediococcus acidilactici*, nisin from *Lactococcus lactis* and carnobacteriocin BM1, carnocyclin A and piscicolin 126 from *Carnobacterium maltaromaticum* are among the few commercial bacteriocins which are approved by the American Food and Drug Administration (FDA). These bacteriocins are generally recognized as safe (GRAS) to be used as food preservatives or food additives. In fact, nisin received approval from the FDA in 1988 for its antimicrobial activity towards *Clostridium* and *Listeria* spores during cheese making [35]. However, these bacteriocins do not show any pathogenicity towards their mother bacteria, due to their specific immune mechanism. Furthermore, their activity or specificity against pathogens can be improved by the bioengineering process. Due to their simple biosynthetic mechanism, their genetic determinants can be manipulated even with few hurdles.

### 3.1. Bacteriocins and Their Classification

Bacteriocins are generally classified based on their bacterial source (whether from Gram-positive or Gram-negative bacteria), molecular size, heat stability, chemical structure, biochemical properties, mechanism of action, etc. (Table 1). Another group of bacteriocins, called archaeocins, are reported from Archaea domain representatives, which have very unique molecular mechanisms to combat the extreme stressed conditions of life. These archaeocins (halocins and sulfolobicins) are produced by *Halobacteriales* and *Sulfolobales* with the characteristics of growth retardation of target pathogens, instead of cell lysis [36,37,38]. Bacteriocins obtained from the Gram-positive LAB group can be divided into four categories, which are class I, class II, class III and class IV. In general, Class I bacteriocins are small (<5 kDa), thermostable, ribosomally synthesized peptides with non-proteogenic thioether amino acids, lanthionine (Lan) and/or methyllanthionine (MeLan), and are therefore called lantibiotics. Lantibiotics undergo extensive post-translational modifications, while class II bacteriocins are small and post-translationally unmodified [36]. Based on their structural and functional differences, these class I lantibiotics are further subdivided into different subclasses (AI, AII and B). Subclass AI consists of positively charged and elongated peptides, which act upon cytoplasmic membranes of sensitive target species forming pores [39]. Subclass B are negatively charged or neutral globular peptides that act by cell wall biosynthesis mechanisms [40]. Nisin from *L. lactis* is one of the best examples of a class I bacteriocin which is used commercially by the food industry. Furthermore, lantibiotics like Lacticin 3147 are two-component lantibiotics that consist of B α-peptide and type A1 β-peptide. This group exhibits synergistic antibacterial activity by the formation of pores in the cell membrane of Gram-positive pathogens [41]. Another group of newly discovered lanthipeptides are lipolantins with avionin residues and N-terminal guanidino fatty acids. Although its mechanism of action is not yet fully understood, microvionin, a representative of the group and extracted from *Microbacterium arborescens 5913*, is reported to have pathogenicity against *Staphylococcus aureus* (MRSA) and *Streptococcus pneumoniae* [42].

Class II bacteriocins are hydrophobic, thermostable peptides with 30–60 amino acids, and are also low in molecular weight (<10 kDa). These classes of bacteriocins contain an amphiphilic helical structure, which helps in the depolarization of the bacterial membrane and cell death of pathogens. Bacteriocins of this class are called non-lantibiotics, as Lan or MeLan is absent [38]. These are further subclassified into subclasses IIa, IIb or IIc, based upon their N-terminal amino acid sequences. Because of their heterogenous chemical structure and broad spectrum of antigenicity for different pathogens, their classification is diverse and critical. Subclass IIa is very much *Listeria*-specific, containing a specific N-terminal peptide chain sequence with one or two α-helixes. Pediocin PA-1, Leucocin A, Sakacin A and P, Enterocin A, etc., are examples of the largest subclass, IIa. There are now 28 different pediocin like class IIa bacteriocins reported with narrow spectrum of activity, higher specificity against *L. monocytogenes* and different hydrophilic or hydrophobic sequences of amino acids in peptide chains called ‘pediocin box’ (conserved C or N terminus) [50]. As far as subclass IIb bacteriocins are concerned, they are heterodimeric, and need two peptides to work synergistically. Their mode of action includes permeabilization of targeted bacterial membrane and decreased intracellular ATP concentration. Lactocin 705 secreted from *Lactobacillus curvatus* CRL705, plantaricin from *L. plantarum*, enterocin from *Enterococcus faecalis*, and lactococcin from *L. lactis* belong to this subclass IIb. Subclass IIc precursor proteins undergo post-translational modifications resultinginto covalent union between carbon (C) and nitrogen (N) terminal portionto createa cyclic structure like circularin A from *Clostridium beijerinckii*, reutericin 6 from *Limosilactobacillus reuteri* [50]. Bacteriocins from different meat sources like enterocin AS-48, carnocycline A and garvicin ML are also some examples of subclass IIc [22]. Another group of unmodified, linear, non-pediocin-like bacteriocins are classified as class IId, which encloses the heterogenous compilation of different antimicrobial peptides from different ecological places. Until now, 31 types of class IId bacteriocins have been discovered, of which the majority are contributed by LAB, e.g., Lactococcin [51].

Class III bacteriocins are large (>30 kDa) heat-sensitive macromolecules [52]. It comprises two subclasses, IIIa or bacteriolysin and IIIb. Lysostaphin and enterolysin A fall under subclass IIIa, showing pathogenicity by cell wall lysis. In a recent study, it has been reported that subclass IIIb (specially Helveticin M) acts by dissipating membrane potential and reducing the intracellular concentration of ATP [53]. Class IV is not considered as a true bacteriocin, due to its complex structural moieties. This group does not meet the antimicrobial properties of bacteriocins. Some microcins and colicins from Gram-negative bacteria *E. coli* belong to this class due to their protein size, microbial targets, antigenicity or immune mechanism [53].

### 3.2. Synthesis and Mode of Action

Bacteriocins are synthesized in ribosomes, and the genetic elements responsible for synthesis and secretion may embrace conjugative transposable elements, genome, plasmid and mobile genetic materials as clusters of operon [53]. Initially, synthesis of small peptides (bacteriocins) from LAB occurs as precursor forms, and further processing and post-translational modification then take place within the cells. The exponential growth phase (during or at the end period) of LAB is crucial for bacteriocins production. The production of bacteriocins depends on the availability of synthesized peptides or pheromones. These peptides normally activate the secretion when it reaches its threshold concentration. After reaching the threshold level, the autophosphorylation of histidine residue takes place due to the activation of transmembrane histidine kinase by bacteriocins, leading to the transfer of phosphate to a response regulator protein [54]. Consequently, bacteriocins attain their mature form after they are transported and cleaved. Mignolet et al. [55] demonstrated that in the absence of BlpAB, the bacteriocin transporter system in *S. salivarius*, the ComRS-regulatedComA, which was homologous to BlpA, could be e identified as a promising candidate for bacteriocin secretion. The genes involved for their expression and export are located near the bacteriocin biosynthesis gene. Among these genes, the first produces the biologically inactive pre-peptide, while the second confers a specific immunity protein towards the producer cell. The third is gene-encoding proteins of the ABC transporter, responsible for exteriorizing the bacteriocin, and the last one helps in bacteriocin exteriorization [22,53]. In general, class II bacteriocins was reported to require a minimum of four genes, including genes for the bacteriocin pre-peptide (e.g., pedA in the case of pediocin PA-1/AcH), the cognate immunity protein (pedB), an ABC-type transport protein (pedD) and a membrane-bound accessory protein (pedC) that is essential for exporting [56]. Nisin acts as autoinducer for its expressions by influencing the two component regulatory systems [53]. After the removal of signal sequence by enzymatic scissor, premature pre-peptides are carried to the extracellular space as mature bacteriocins.

Bacteriocins (specially cationic) commonly target anionic bacterial cell surface components such as phosphatidylethanolamine, phosphatidylglycerol, lipopolysaccharide, lipoteichoic acid and cardiolipin [57,58]. Bacteriocins bind to specific receptors of target pathogenic and non-pathogenic bacteria’s cell wall, and subsequently either kill them or reduce their pathogenicity by different adjuvant mechanisms [59]. Bacteriocins are normally found to have positively charged peptides with hydrophobic regions that commonly interact electrostatically with the negatively charged bacterial cell surface, whereas the hydrophobic regions traverse the lipid bilayer.

**Figure 1:**
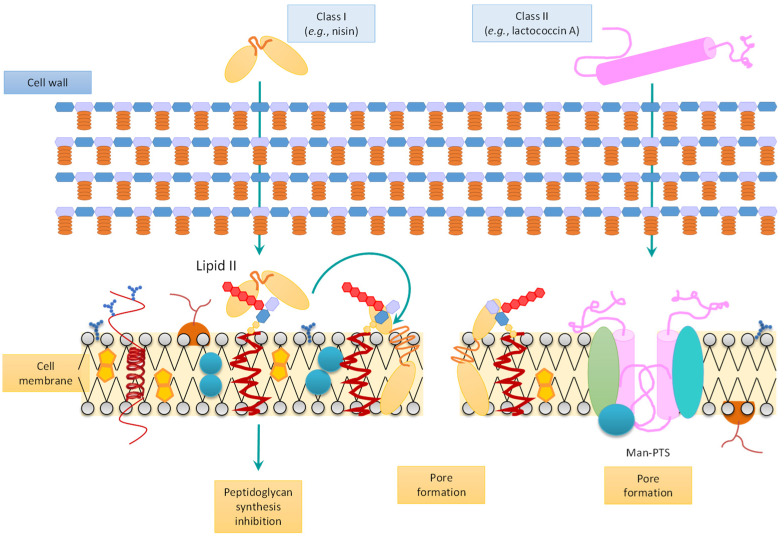
Mechanism of action of bacteriocins on Gram-positive bacteria. Adapted from da Costa et al. [22].

In the case of Gram-positive bacteria, bacteriocins may work by two different mechanisms (Figure 1). In the case of the class I model, bacteriocins inhibit the synthesis of components related to bacterial cell wall and lipid II in the cell membrane. Meanwhile, in the class II model, the formation of ion-selective pores in the cell membrane takes place through pore forming receptors in the mannose-phosphotransferase system, which causes dissipation of the proton motive force and the depletion of intracellular ATP, leakage of intracellular substrates, and eventual death [53].

Bacteriostatic activity of bacteriocins depends on dose, degree of purification, growth phase, pH, temperature or other antimicrobial substances present to alter the cell wall integrity. Among many, nisin and pediocin are popularly known bacteriocins with a strong antimicrobial spectrum that inhibits the activity and growth of spoilage and pathogenic bacteria. Although their use is approved by the competent authority for food and pharmaceutical sectors, they are not so promising towards gram-negative bacteria causing foodborne diseases. It has been reported that the use of chemicals (e.g., organic acids, essential oils, EDTA) or physical (e.g., temperature, pulsed electric field, pH, high hydrostatic pressure) stress to destabilize the outer membrane of the cell wall could be effective, and increase the activity of bacteriocins for Gram- negative bacteria [22,47].

### 3.3. Microbial Resistance

LAB strains that produce bacteriocins develop an immune mechanism against their own metabolites, leading to bacteriocin resistance. However, the resistance against self-virulence is attained through different mechanism of actions [59,60]. Various types or classes of bacteriocins act by tethering to an important precursor, lipid II present in bacterial cell wall, different enzymes (permease mannose phosphotransferase, zinc-dependent membrane-bound proteases), and transporters such as maltose ABC transporter [61]. They exert their effect by first influencing the membrane receptors, which serves as docking molecule for bacteriocins. For example, the maltose ABC transporter complex is indispensable for Garvicin ML as receptors, and its absence may lead to development of resistance in the *Enterococcaceae* family [61]. Any alternation in the chemical composition of teichoic acid decreases the negative charge of the bacteria cell wall. It is well known that teichoic acid is responsible for the highly negative charge to the cell wall of gram-positive bacteria, but the coupling of D-alanine to lipoteichoic acid (D-alanylated) results in the replacement of negative charges with positive charges, and this change probably prevents bacteriocins to tether lipid II in cytoplasmic membrane [62]. Resistance in daptomycin of *Staphylococcus aureus* has been partially due to the above coupling effect of D-alanine to teichoic acid [63,64]. Reports also indicated that D-alanylation of lipoteichoic acid of bacterial cell wall provides protection against bacteriocins and other different antibacterial peptides. The incorporation of D-alanine into the lipoteichoic acid of a bacterial cell wall is facilitated by the *dltABCD* operon system. Even many bacteria cells could be able to alter anionic phospholipids with L-lysine to yield a basic phospholipid known as lysophosphatidyl glycerol and develop a net-positive charge on the bacterial cytoplasmic membrane, which can protect from antimicrobial peptides or bacteriocins, including the lipopeptide daptomycin. The high proportion of altered and desaturated acid, as well as short acyl chain fatty acids in the bacteriocin-resistant variants or microorganism, is attributed to the increased rigidity of the membrane and less fluidity, limiting the membrane penetration by bacteriocins into the cell [60,65]. Resistance can also be acquired by natural transformation with free DNA that codes for immunity genes present in the genomic frame of bacteriocins [66].

### 3.4. Purified or Semi-Purified Bacteriocins

Being a natural preservative for meat or meat products, the applicability includes either a whole bacteriocin-producing strain in food or the incorporation of purified or partially-purified bacteriocins as additives [67]. In the trademark Nisaplin^TM^, for example, nisin (2.5%) is a water-soluble, thermostable component [68], but shows a strong bactericidal activity against major Gram-positive food pathogens such as *B. cereus* and *L. monocytogenes*. The crude form of bacteriocin (i.e., MicroGARD^TM^) is effective against *S. aureus* and *L. monocytogenes*, and also against Gram-negative strains such as *Pseudomonas* and *E. coli* [67,69]. Some producers of protective bacteriocins (sakacin) like *L. sakei* and *Staphylococcus xylosus* are employed to control *L. monocytogenes* in meat products stored in vacuum packing. Other cultures like *P. acidilactici*, *L. curvatus*, *L. plantarum*, *Staphylococcus carnosus* etc., are available commercially under different names (e.g., Bactoferm^TM^ B-FM, Bactoferm^TM^ F-LC, ALCMix 1) for meat preservation, especially against *L. monocytogenes* [68].

Purified bacteriocin plantaricin (from *L. plantarum*), isolated from local beef of Indonesia, and applied at a 0.3% level as a nitrite substitute, showed positive results against *E. coli* in meatballs stored under refrigerated conditions for 6 days, without altering the nutritional and physical changes [70]. This is important for the meat industry, as WHO has sounded the alarm on the use of nitrate or nitrite in cured meat products as their carcinogenic intermediate—therefore, bacteriocin could be a potential alternative. Again, partially purified bacteriocin, BacFL31 isolated from *Enterococcus faecium*, showed positive results against *L. monocytogenes*, *S. typhimurium* and *S. aureus* in ground turkey meat, and enhanced the shelf life to up to 14 days at 4 °C [71,72].

Other than commercialized bacteriocins, researchers have also designed multi-bacteriocin producing microorganisms to obtain enhanced antimicrobial properties to control foodborne pathogens. The molecular bioengineering of bacteriocins or manipulation in amino acid sequences could be used to broaden the antimicrobial spectrum of peptides, or to enhance the delivery and release rate in the food system. In the above direction, Balay et al. [35] developed an analogue of leucocin A by replacing asparagine in position 17 with another amino acid, leucin, to obtain the leucin N17L variant. The researchers then designed a study on poultry meat to check whether *Listeria* counts could be controlled in a better way or not. Unfortunately, the new variant showed no better results compared to the original one. Among all the purified bacteriocins, nisin is the most popular, and the only one commercialized and approved as a food additive [73]. Although many bacteriocins are available, low yields and difficulties in purification limit their use on a large scale. To overcome this challenge, considerable progress has now been made in peptide synthesis of class II and S-glycosylsated bacteriocins by chemical approach, which are cost effective compared to recombinant technologies [74].

## 4. Bacteriocins Isolated from Meat and Meat Products

Based on the novel functionality of LAB and their metabolites, several experiments are being conducted by researchers from different countries (Table 2). For example, Gomes et al. [75] isolated almost 60 LAB from raw beef, processed meat, viscera, poultry, pork and fish. Though none of these showed bacteriogenic activity, the most common strain was of *Enterococcus* sp. Dal Bello et al. [26] also found some LAB strains in ground meat and fermented meat products, of which *Lactococcus* and *Enterococcus* were predominant, and had the bacteriocinogenic properties against *L. monocytogenes* and *S. aureus.* Castro et al. [76] identified *L. curvatus*/*sakei* ACU-1, sensitive to trypsin and proteinase K, in different fermented sausages. This bacteriocin exhibited heat stability, and its production was modified by the presence of suitable surfactants and different concentrations of NaCl. Another study conducted by Fontana et al. [77] allowed to isolate 115 meat-borne LAB with anti-listerial properties. These were *L. sakei*, *L. curvatus*, *L. plantarum*, *Enterococcus faecium* and *P. acidilactici*. These isolates showed bioprotective properties for the control of *L. monocytogenes*. From these findings, it can be concluded that not only bacteriocins, but also live cultures, have biopreservation properties by producing fermented meat products, positively affecting organoleptic properties.

### 4.1. Bacteriocins Used in Meat and Meat Products

LAB or their active metabolites can be incorporated in any kind of foods, such as dairy products, meat or meat products, or even fruits and vegetables. The purpose of using these biopreservatives in food products is not only to control foodborne organisms, ensuring public health safety, but also to meet consumers’ demand of using natural preservatives. LAB or their active peptides can kill the target pathogens by disrupting their cell membrane [106], and can be used as a potent solution to reduce microbial resistance to antibiotics. Direct application of purified or semi-purified active peptides of LAB into product formulation during processing, or the live cultures into products as part of fermentation for in-situ production of bacteriocins, are the normal practices in vogue in the meat processing industry. Other strategies applied to incorporate LAB or bacteriocins into meat or meat products are antimicrobial active packaging by adsorption or antimicrobial coatings. Again, metabolites of LAB such as bacteriocins, lactic acids, including various organic acids, are utilized as antimicrobial component in packaging or films to actively reduce the growth of spoilage microorganisms in foods [107].

De Martinez et al. [108] applied nisin and lactic acid spray (1.5%, 25 °C) to poultry and bovine carcasses to reduce the aerobic, *E. coli* or coliform counts. However, the main drawback of using nisin alone in meat products is its low solubility, enzymatic destruction and inefficiency of inhibition of pathogenic microbes. After nisin, pediocin (*P. acidilactici*) is considered the most effective bacteriocin for its strong antimicrobial action against *Listeria* spp. Their use in the meat industry can be made effective with low pH, or with the addition of lactate or other organic acids [109]. It has been reported that pediocin and nisin reduced *Lactobacillus sakei* counts in sliced ham that was vacuum-preserved. *P. acidilactici* MCH14 and *Pediococcus pentosaceus* BCC3772 (producer of pediocin PA-1, AcH) were found to reduce *L. monocytogenes* counts in different fermented meat products without altering any sensorial characteristics [110,111]. Swetwiwathana et al. [112] observed that *P. pentosaceus* strain (pediocin producer) inhibited the growth of *Salmonella anatum* in fermented meat sausage. The combinations of different bacteriocins have also improved the inhibition of foodborne pathogens. For example, sakacin G and sakacin P, produced by *L. sakei* CWBI-B1365 and *L. curvatus* CWBI-B28, respectively, showed promising results in inhibiting the growth of *L. monocytogenes* in chicken meat and beef [113]. Similarly, semi-purified bacteriocin BacTN635 produced by *L. plantarum* sp. TN635, and isolated from meat, reduced the proliferation of *Listeria* and other spoilage microorganisms in beef and chicken breast in refrigerated storage conditions [114].

Bacteriocinogenic LAB strains also may exert good results as a probiotic. Normally, probiotic microbes with GRAS status show a beneficial effect in the gastrointestinal tract by colonizing the intestinal mucus. Apart from being tolerant to pepsin and pH or other enzymes, they suppress the activity of enteropathogenic bacteria. Therefore, fermented meat products with probiotic cultures are of interest for their health benefits to consumers.

**Figure 2:**
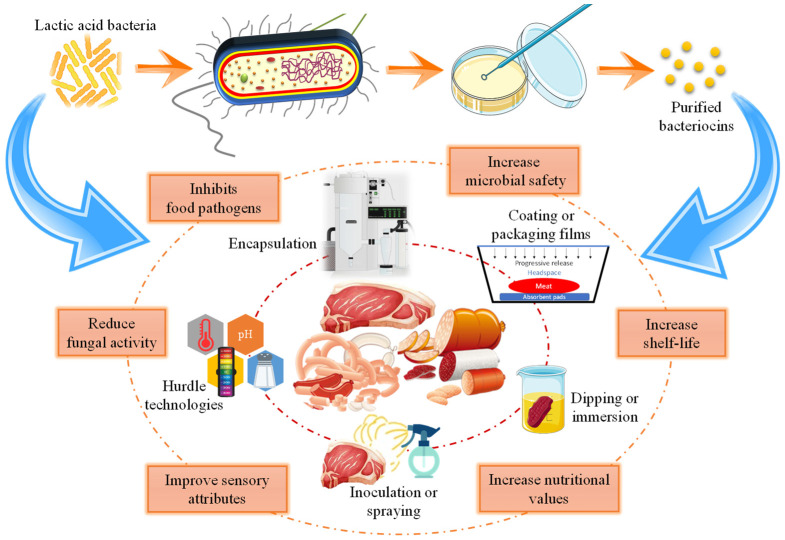
Biopreservative effect of lactic acid bacteria and bacteriocins on quality and safety of meat and meat products.

Recently, novel bacteriocins are being purified and characterized from various LAB for possible application as a biopreservative in different food systems (Figure 2). Lu et al. [91] reported a positive inhibition of *E. coli* and *S. aureus* in beef meat when stored at 4 °C through a cell envelope-associated mechanism of the novel bacteriocin BM1029, isolated from *Lactobacillus crustorum* MN047. In another study, Bac-IB45, a purified bacteriocin extracted from *L. plantarum* KIBGE-IB45, was used as a biopreservative to enhance the shelf life of meat products. Results showed that Bact-IB45 not only inhibited the growth of *L. monocytogenes* ATCC-7644, but also maintained the original color, pH and texture of meat samples during the 14 days of storage at refrigerated temperature [115]. Bacteriocin produced from *P. pentosaceus* strain 2397 was also found to inhibit the growth of *S. aureus*, *L. monocytogenes* and *E. coli*, and maintained the quality of meatballs during storage [116]. It has also been reported that the antimicrobial action of bacteriocin from *P. pentosaceus* strain 2397 can be significantly increased by incorporating peptone, beef extract, ammonium sulphate, lactose or tween into the de Man, Rogosa and Sharpe broth (MRSB). In a different study, chicken breast strips marinated with anti-listerial beer containing leucocin C, extracted from a probiotic yeast *Saccharomyces boulardii*, were reported to have significant inhibition of growth of *L. monocytogenes*, and beer was able to maintain anti-listerial activity for a period of 38 days [89]. Similarly, metabolites (Sak-59) with bacteriocin-like activity extracted by the probiotic *L. sakei* B-RKM 0559 strain from Kazy, a traditional horse meat product, can be used as a potential antimicrobial for the preservation of foods. This strain exhibited strong inhibitory activity against major food pathogens such as *L. monocytogenes*, *S. aureus*, *Serratia marcescens* and *E. coli* [100].

### 4.2. Bacteriocins and Hurdle Technology

There are several reports available on control of foodborne outbreaks and extension of shelf life of products by applying the hurdle concept. Instead of using alone, LAB or bacteriocins can be combined with any natural antimicrobial substance to produce a synergistic effect. Khalili Sadaghiani et al. [82] conducted a study with *Limosilactobacillus reuteri* and *L. plantarum* individually and with 1% garlic extract in raw beef stored for 12 days at 4 °C. The LAB strains and garlic extract, when used together, reduced up to 1.5 log cfu/g of *L. monocytogenes*. Moreover, their sensory attributes were quite favorable from a consumer point of view. A better result was observed when chelating agents such as Na_2_EDTA were used in combination with LAB strains *L. curvatus* and *L. lactis* to control *E. coli* O157H7 and coliforms in ground beef patties stored for 9 days at 5 °C [117]. In this hurdle concept, chelating substance made the outer cell membrane of Gram-negative bacteria more permeable to hydrophobic peptides of bacteriocins. Different investigations have reported a promising reduction of *Salmonella*, *E. coli* and *L. monocytogenes*, when bacteriocins were combined with different chelators like nitrates, citrates and/or EDTA [39,117]. Different natural anti-microbial substances or antioxidants with bacteriocins or bacteriocin-producing LABs are also reported to reduce foodborne outbreaks and improve nutritional or sensory properties of meat products as well. The use of the essential oil *Mentha piperita* at a 0.25–0.5% level showed a strong biopreservative action in combination with semi-purified bacteriocin BacTN635 from *L. plantarum* at 500–1000 AU/g. This combination not only reduced lipid oxidation and improved sensory parameters, but also extended the shelf life of chill-stored minced beef by 7 days [118]. Different organic acids like acetic acid or lactic acid, used at 2.5%, in combination with bacteriocins produced by *L. curvatus* CRL705 or *L. sakei* as a dipping solution, showed a positive outcome by reducing pathogen counts in frankfurters stored at 10 °C for 36 days [81]. However, it should be borne in mind that all randomly selected combinations may not show synergism, so it is crucial to only combine them after knowing their mechanical properties.

Modified atmosphere packaging (MAP), vacuum packaging and freezing temperature can also be a part of the hurdle concept for the biopreservation of meat with bacteriocins [119]. Siragusa et al. [120] found a positive result when *B. thermospecta* bacteriocin was used to control pathogen population from log_10_ 7 to log_10_ 3 in meat in a plastic container. Budde et al. [121] observed that bacteriocin synthesized from *Leuconostoc carnosum* was able to reduce the population of pathogen in meat products stored and vacuum-packed for 21 days. In another study, *Leuconostoc pseudomesenteroides* reduced *L. monocytogenes* and *C. jejuni* at noticeable levels in chicken burgers stored at −18 °C for 48 h, when combined with a 50% CO_2_/50% O_2_ MAP [119]. Casquete et al. [78] noticed that the combination of the antimicrobial properties of *L. sakei* and commercial LAB strains under MAP conditions (20% CO_2_/80% N_2_ and 40% CO_2_/60% N_2_) could reduce 1–2 log cycles of *L. innocua* effectively in a cured smoked pork loin. Segli et al. [122] stated a positive reduction of exopolysaccharide producer *Latilactobacillus sakei* CRL1407 in vacuum-packaged meat discs at 4 °C by bioprotective cultures of *Lactobacillus acidophilus* CRL641 and *Latilactobacillus curvatus* CLR705.

A synergistic effect was also noticed while controlling the growth and survivability of *L. innocua* (up to 2 log cfu/g), when applying treatments with pediocin bacHA-6111-2 and high hydrostatic pressure (300 MPa at 10 °C for 5 min) on a Portuguese fermented product [97]. Another study conducted by Orihuel et al. [123] reported reduced *Listeria* counts of up to 2 log cfu/g in a beef sausage model using a hurdle with bacteriocinogenic strain of *Enterococcus mundtii* and curing derivatives such as NaCl, NaNO_2_, ascorbic acid, sucrose and glucose. Similarly, in combination with extracts from *Murraya koenigii* berries, pediocin from *P. pentosaceus* could reduce *Listeria* counts effectively in goat meat emulsion [103]. Even nisin and *Salmonella* bacteriophage, when applied to fresh pork meat, controlled *S.* Typhimurium up to 1 log cfu/g [124]. Oregano essential oil, in combination with nisin, could also show a promising result in controlling *E. coli* counts in meat products [125]. This would be due to the presence of active components like carvacrol and thymol, which destabilize the outer membrane of bacterial strain to facilitate the antimicrobial action of nisin. Bacteriocins with commercial antimicrobials can sometimes improve the overall antimicrobial activity. For example, the application of vancomycin in combination with bacteriocin from *L. plantarum* ST8SH had a satisfactory performance regarding the formation of biofilm produced by *L. monocytogenes* strains [126].

## 5. Active Packaging and Bacteriocins

The direct application of bacteriocins in a meat matrix may inactivate the potency of antimicrobial substances. Therefore, other methods such as intelligent or active packaging techniques with antimicrobial compounds are also employed. In general, the incorporation of bacteriocins into packaging materials are mostly done by soaking, blow processing, extrusion, direct contact, coating, etc. By this process, the controlled or slow release of active peptides/bacteriocins that act on the surface of products and control foodborne spoilage microorganisms is produced. Therefore, intelligent packaging with antimicrobial compounds has a promising future, particularly in the meat industry, as this technique is safe, easy to handle, and above all a chemical-free preservation method [1,127,128]. However, there are limitations regarding its direct use on plastic film, due to its hydrophobic nature [129].

Various reports have highlighted the use of nisin and low density polyethylene, cellophane, and chitosan, showing an efficient inhibition of potential pathogens including *L. monocytogenes* and *B. thermosphacta* in different meats (raw beef, sliced ham, pork) [130,131]. However, the application of nisin into meat products has a few drawbacks. Apart from having low solubility, nisin also interacts with other food ingredients, including phospholipids and emulsifiers [132]. The use of LAB metabolites with various packaging materials in the form of films or coatings allows the inhibiting of the growth of microorganisms. In this regard, the use of plantaricin BM-1 in combination with LDPE and PE films have been reported to suppress the growth of pathogens like *L. monocytogenes* and *S. aureus* in raw meat [133]. The application of plantaricin BM-1 and chitosan, in combination with different multilayered plastic films such as polyethylene terephthalate (PET)/polyvinylidene chloride (PVC) or retort casting polypropylene, also inhibited the pathogenic microbial growth in meat stored at a refrigerated temperature [134]. In another study, Trinetta et al. [135] applied sakacin A, along with a biodegradable packaging material ‘pullulan’ produced by *Aureobasidium pullulan* in turkey breast meat. The controlled release of sakacin A into the food matrix resulted in reducing the *L. monocytogenes* counts by 3 log cfu/g after 21 days of storage under chilled conditions, compared to sakacin A applied directly to turkey breast, which could reduce 2 log cfu/g during the same period.

Antimicrobials can protect against cross-reactions with food ingredients when they are applied with packaging materials. A similar result was found in raw sliced pork stored at refrigeration temperature for 14 days with a biocomposite film of polylactic acid and sawdust particles incorporated with pediocin PA-1/AcH. The use of the diffusion-coating technique caused a reduction of approximately 1.5–2 log units of *L. monocytogenes*, which could be due to the fact that it aided a better adsorption of the bacteriocin [129]. More advanced technology, such as encapsulation, can maintain and enhance the bioprotective nature of LAB metabolites from the risk of degradation by food components, and their slow and controlled release acts more precisely in the inhibition of pathogens. Ghabraie et al. [136] encapsulated nisin and other antimicrobial compounds like essential oil, nitrite and organic acids into alginate-cellulose nano-crystal microbeads, and applied it in sausages. This technique could reduce microbes up to 2.6 log cfu/g after one week of storage under refrigerated conditions.

## 6. Commercialization and Toxicity

LAB or their metabolites have been used in fermented food products since time immemorial. Research conducted in this area has also proved the efficacy of LAB and their metabolites as good preservatives or antimicrobial substances. Although many bacteriocin-producing strains are available, their acute, chronic or sub-chronic toxicity, sensitivity and cross-resistance studies, or any undesirable effects on consumers, are not documented. As per the Antimicrobial Peptide Database, although more than 335 bacteriocins are recorded [137], toxicity data of very few bacteriocins has been described. Only nisin is approved for commercial use by Codex Alimentarius, with a maximum intake of 2.9 mg person^−1^ day^−1^ [138]. Even though several bacteriocins are available, the safe dose limit of antimicrobial substances is not recognized. This is important, as excessive consumption of bacteriocin may lead to the development of certain unspecific bactericidal effects, which could be hazardous to consumers. Therefore, the stability, cytotoxicity and permeability of bacteriocins must be evaluated using in-vitro and in-vivo animal models before their application in different food matrices.

In a recent study conducted using in-vitro simulations, it has been reported that bacteriocins (nisin and plantaricin 423) are capable of migrating across gastrointestinal epithelial and vascular endothelial cells [139]. In order to protect the bacteriocins from degradation in the upper gastro-intestinal tract, the encapsulation [140] or incorporation of bacteriocins in starch-based matrices may help in overcoming such limitations [141] (2018). Further studies are needed to determine the optimum concentration and dosage of bacteriocins required before the possible use of these peptides as an alternative to antibiotics/chemical preservatives in different matrices.

Concisely, in order to obtain approval tags from the safety and standards authorities, bacteriocins need to undergo several toxicological tests and clinical trials, and be evaluated for parameters such as safe dose and acceptable daily intake before their commercial use in food products. Therefore, despite being potent antimicrobial components, or their ability in food conservation, many bacteriocins still have a rare commercial applicability. Hence, more advanced research trials are required in this field, keeping consumers’ safety in mind.

## 7. Conclusions and Future Prospects

Since food quality and safety issues are becoming increasingly global concerns, more attention is now paid to overcome them by inhibiting the growth of pathogenic and spoilage microorganisms in food products. In this regard, LAB and antimicrobial metabolites have been widely studied since their origin over the last few years to prevent spoilage, thereby maintaining the quality and safety aspects of meat and meat-based food products. However, bacteriocins—the active metabolites of LAB—are still underutilized despite their novel biopreservation activity on meat and meat products. Various limiting factors such as a narrow antimicrobial spectrum, higher cost of production, low yield and requirement of a higher dosage are some of the factors that restrict their use in the commercial sector. Therefore, more analytical, and advanced research is required to overcome these hurdles before the realization of their full potential in meat processing and biopreservation. For this, research should be targeted towards the development of cost-effective methods to optimize the production and purification processes. In recent days, various strategies such as combining bacteriocins of two classes through the genetic bioengineering approach or conjugating the existing bacteriocin-producing strains with nanoparticles in combination with the use of non-thermal techniques like high hydrostatic pressure, irradiation, pulsed electric fields, ultrasound, and hurdle concepts, etc., are employed to exert synergistic effects on food protection and overcome the bottlenecks. These processes not only reduce pathogenicity of spoilage microorganisms through their enhanced antimicrobial spectrum and efficacy, but also lower antimicrobial resistance incidence, apart from improving the sensorial characteristics of meat with an enhanced shelf life. However, all proposed additives should be toxicologically tested (both in vitro and in vivo) and evaluated for their accumulative, synergistic, and potential effects prior to seeking approval from the regulatory framework. As bacteriocins are going to be added in food matrix intended for human consumption, it is pertinent to study the absorption rate, distribution pattern, and metabolic activities of these biological molecules, as well as to establish concentration thresholds (dosage) to ensure that upon application, these additives do not alter the physicochemical, nutritional values and sensory attributes inherent to the products. These advancements will surely pave the way for commercial applications of LAB and their metabolites, particularly bacteriocins in the processed food industry, including the meat sector, in the coming days.

## Figures and Tables

**Table 1:** Different classes of bacteriocins and their characteristic features.

Class Type	Producing Strain	Subclasses	Characteristic Features	Major Bacteriocins	Ref.
Class I (Lantibiotics)	*Lactococcus lactis* subsp. *lactis*, *Staphylococcus epidermidis*, *Streptococcus mutans* *Streptococcus salivarius* *Lactococus uberis* *Staphylococcus gallinarum*	Sub-class AI, AII (Linear and combined molecules)	✓ Elongated, flexible, positively charged peptides ✓ Acts on cell membrane and damages the cell membrane by pore formation	Nisin, Lacticin 481, Enterocin W, Lactocin, Epidermin, Mutacin B-Ny266, Gallidermin, Mersacidin, Salivaricin A, Lacticin 3147	[38,43,44]
		Sub-class B (Globular molecules)	✓ Normally compact, with globular peptide chains either negative or no net charge ✓ Disrupts enzymatic reactions in target species		
Class II (Non-lantibiotics)	*Lactiplantibacillus plantarum*, *Lactobacillus acidophilus*, *L sakei*, *Streptococcus uberis* *Leuconostocmesenteroides* *Pediococcusacidilactici*, *Enterococcus faecalis*, *Carnobacteriumpiscicoda*, *C. Divergens*	Class II A (Anti-listerialpediocine bacteriocins type)	✓ Small antimicrobial peptides (<10 kDa) with heat-resistant properties ✓ Comprises pediocin like peptides ✓ Exhibits anti-listerial activity	Sakacin A, Pediocin PA-1/Ach, Carnobacteriocin X	[43,45]
		Class II B	✓ Composed of two different peptides	Lactococcin G, Lactacin F Plantaracin EF and JK	
		Class II C (Other bacteriocins)	✓ Circular peptides, N-terminal and C-terminal covalently linked	Acidocin B, Carnobacteriocin A, Divergicin A, Enterocin A, P	
Class III (Large peptides)	*Lactobacillus helveticus*, *L. bulgaricus* *Lacticaseibacillus casei*	Class IIIA (Lytic enzymes)	✓ Larger size peptides (>30 kDa), heat-sensitive ✓ Capable of binding to the receptor	Helvetic in *Lactobacillus helveticus*, HelveticinV-1829, Enterolysin A	[43,46,47]
		Class IIIB (Non-lytic proteins)	✓ Hampers glucose uptake and ✓ Disturbs cell membrane potential	Caseicin 80 Helveticin J	
Class IV Cyclic bacteriocins	*E. faecalis* *L. helveticus 481*		✓ Protein with chemical moieties (N- and C-terminus) ✓ Attaches to the cell membrane and facilitates cell lysis	Enterocin AS-48 Glycocin F	[48,49]

**Table 2:** Effect of LAB and bacteriocins on microbial, physical and sensory quality of meat and meat products.

Biopreservative Agent	Product	Major Findings	Ref.
LAB cultures (*Lactobacillus sakei* ST153) in combination with MAP (either 20% CO_2_/80% N_2_ or 40% CO_2_/60% N_2_) in RTE sliced ‘lombo’, a traditional cured-smoked pork loin	Microbial growth and sensory attributes of cures-smoked pork loin stored at 5 °C for 124 days	✓ The combined treatment reduced *L. innocua* 2030 c counts of ‘lombo’ to 5.0 log CFU/g for 120 days ✓ Maintained sensory quality and safety of cured smoked pork products with respect to *Listeria* spp.	[78]
Bacteriocin-like inhibitory substances (BLIS) from *Pediococcus pentosaceus* ATCC 43200 in artificially contaminated RTE pork ham	Physico-chemical and antimicrobial activity of BLIS against *Listeria seeligeri* NCTC11289	✓ Growth of *L. seeligeri* NCTC11289 inhibited (counts from 1.74 to 0.00 log CFU/g) for 6 days ✓ Treated ham samples recorded lower weight loss (2.7% vs. 3.0%) and lipid peroxidation (0.63 vs. 1.25 mg MDA/kg) compared to control ✓ BLIS did not influence coloration such as redness, and yellowness, including discoloration of ham samples	[79]
LDPE film coated with sonorensin, a bacteriocin from *B. sonorensis* MT93	Chicken meat pieces spiked with 2 mL suspension of *Listeria monocytogenes* and *Staphylococcus aureus* at 1.5 × 10^6^ CFU/mL and stored at refrigerated temperature up to 15 days	✓ Sonorensin effectively controlled *L. monocytogenes* and *S. aureus* ✓ No spoilage observed in meat packed with sonorensin-coated films even after 15 days, in comparison to control samples (4 days) ✓ Sonorensin could be used as a promising antibiofilm agent natural food biopreservative	[80]
Semi purified bacteriocin BacFL31 (secreted by *Enterococcus faecium* sp. FL31) at 200 and 400 AU/g	Physicochemical microbial and sensory attributes of turkey meat stored under refrigerated conditions for 14 days	✓ BacFL31 was effective in suppressing *L. monocytogenes* and *Salmonella* Typhimurium in meat ✓ Treated samples exhibited lower pH, % Met-Mb, and TBARS values (*p* < 0.05) than the control sample ✓ Treated meat samples had extended shelf life and improved sensory attributes	[71]
Frankfurters in dip solution containing semi-purified bacteriocins (*Lactobacillus* *curvatus* CRL705 or *L. sakei)* in combination with acetic acid or lactic acid at 2.5%	Microbial and sensory studies of vacuum packaged beef frankfurters stored at 10 °C for 36 days	✓ Combination treatment reduced pathogens in beef frankfurters to below detection level from the sixth day until the end of storage ✓ Treated meat products had extended shelf life ✓ Treated frankfurters had higher flavor intensity and darker color without having any negative impact on flavor, and overall product acceptability of up to 22 days of storage at 5 °C	[81]
Garlic extract (1%) in combination with *Limosilactobacillus reuteri* (G-LR) and *Lactiplantibacillus plantarum* (G-LP)	Effect of combination treatment on physico-chemical microbial and sensory characteristics in ground beef samples stored at refrigerated temperature up to 12 days	✓ G-LR or G-LP treatment reduced the *L. monocytogenes* count (2.13 and 2.57 log) in ground beef samples ✓ G-LP was more effective and significantly (*p* < 0.05) inhibited aerobic mesophilic bacteria by 1.64 log cycle and *L. monocytogenes* counts by 1.44 log cycles ✓ G-LP treated samples had significantly (*p* < 0.05) lower lipid oxidation and increased shelf life	[82]
Bacteriocinogenic activity of *Lactobacillus acidophilus* PTCC 1643 and *Bifidobacterium animalis* ssp. Lactis BB-12 PTCC 1736	Anti-microbial activity of bacteriocins on fresh red beef minced meat stored at refrigerated temperature up to 14 days	✓ *L. acidophilus* showed a significant biopreservative effect against two pathogenic bacteria, *S. aureus* and *S.* Typhimurium in minced meat	[83]
Surface application *L. curvatus* L442 and *L. lactis* subsp. cremoris ATCC 14365 bacteriocin (0.6 g/bag) to hotdogs inoculated with *L. monocytogenes* (4 log CFU/hot dog)	Anti-listerial activity of bacteriocin on vacuum-sealed hot dogs stored under refrigerated conditions for 28 days	✓ Bacteriocins significantly decreased *L. monocytogenes* count from the surface of hot dogs ✓ Bacteriocins effective in reducing *L. monocytogenes* count to 2 log cfu/hot dog during the storage period	[84]
Lactococcin BZ bacteriocin (produced by *L. lactis* spp. lactis BZ)	Microbiological quality of fresh beef treated with lactococcin BZ (200–2500 AU/mL) and kept at 4–5 °C for 12 days	✓ Lactococcin (at 2500 AU/mL) decreased the log cycle count of mesophilic (4.87), psychrotrophic (3.50) and lactic acid bacteria (3.94) at the end of storage compared to control sample ✓ Lactococcin BZ (1600 AU/mL) exhibited a very strong anti-listerial effect with reduced count of *L. innocua* from 6.04 log CFU/g to undetectable levels in fresh meat on the sixth day of storage	[85]
*L. curvatus* 54M16 (Sakacins X, T and P)	Fermented sausage	✓ Treated sausages had reduced pH and microbial count (Staphylococci and Enterobacteriaceae), altered content of free amino acids when compared to the control	[86]
Immersion of beef meat in BM1829 bacteriocin derived from *Lactobacillus crustorum* MN047	Anti-bacterial effect of bacteriocin and its potential use as a preservative of beef meat stored at refrigerated temperature	✓ Bacteriocin BM1829 exhibited broad-spectrum inhibitory activity against both Gram-positive and Gram-negative bacteria ✓ Significant reduction in the number of *Escherichia coli* and *S. aureus* cells in beef meat treated with BM1829 was observed ✓ Bacteriocin extended the shelf life of beef meat up to 10 days, and could be used as antimicrobial bio preservative	[87]
Nisin derived from *L. lactis*	Microbial quality of minced beef stored under refrigerated temperature for 15 days	✓ Nisin inhibited food-borne pathogens (*L. monocytogenes* and *Bacillus cereus*) and improved safety of minced beef under refrigerated storage conditions	[88]
Bacteriocin leucocin C (strain of *Saccharomyces boulardii* CNCM I-745)	Anti-listeria activity of bacteriocin on chicken breast strips marinated overnight in beer spiked with *L. monocytogenes*	✓ Reduced viable cells of *L. monocytogenes* by about 1.6 log from 2.2 × 10^7^ CFU/g on day 24, and 2.2 log from 1.8 × 10^5^ CFU/g on day 38	[89]
Novel bacteriocin (XJS01) from *Ligilactobacillus salivarius* strain CGMCC2070	Raw chicken breast piece marinated with beer brewed with bacteriocin	✓ Bacteriocin inhibited the *S. aureus* strain 2612:1606BL1486 isolated from chicken meat ✓ Could be used for control of *S. aureus* in foods in either planktonic or biofilm states	[90]
Novel bacteriocin BM1300 produced by *L. crustorum* MN047	Effect of bacteriocin on beef meat sprayed with *S. aureus* and *E. coli* (5 mL, 10^6^ CFU/mL) and stored at refrigerated temperature for 10 days	✓ Bacteriocin exhibited antibacterial activity through the inhibition of biofilm formation and the disruption of cell cycle distribution ✓ BM1300 showed better antimicrobial effect against *E. coli* than S. *aureus* and improved the microbiological quality of beef meat	[91]
Novel bacteriocin BM1122 derived from *L. crustorum* MN047	Anti-bacterial activity of BM1122 in fresh raw beef meat stored under chilled conditions for 10 days	✓ Novel bacteriocin BM1122 exhibited a broad inhibitory spectrum against selected Gram-positive and Gram-negative bacteria ✓ BM1122 had bactericidal efficiency on both *S. aureus* and *E. coli* in beef meat stored under refrigerated conditions for 10 days	[92]
*Lacticaseibacillus paracasei* (LP) bacteriocin	Effect of bacteriocin on microbial and cooking qualities, physico-chemical parameters of raw and roasted pork	✓ Spraying the raw meat sample was the most efficient method of application compared to rinsing and dipping ✓ Cooking qualities of meat and degree of preference were not affected by LP treatment ✓ LP significantly (*p* < 0.05) reduced the growth of Staphylococci in raw pork after 8 h under laboratory conditions ✓ LP was effective in significantly (*p* < 0.05) inhibiting the aerobic bacteria and coliforms growth after 3 and 6 h, respectively	[93]
*L. curvatus* UFV-NPAC1 or its partially purified bacteriocin at 12.5 mg/g and 6.25 mg/g	Inhibitory activity of bacteriocinogenic strain on *L. monocytogenes* in fresh pork sausage stored at 7 °C for 10 days	✓ *L. curvatus* UFV-NPAC1 was effective in controlling *L. monocytogenes* growth in fresh sausage compared to its partially purified bacteriocin at both tested concentrations	[94]
Purified pediocin AcH/PA-1, produced by *P. pentosaceus* OZF	Anti-listeria activity of bacteriocin in chicken meat products radiated and inoculated with *L. monocytogenes* (10^5^ CFU/g) and stored under refrigerated conditions for 14 days	✓ Purified pediocin significantly reduced listeria counts (3.8 log CFU/g) in chicken meat samples compared to the control	[95]
*P. acidilactici* HA-6111- 2 or its bacteriocin, pediocin PA-1 (1280 AU/g) alone or in combination with mild HHP (300 MPa, 10 °C, 5 min)	Anti-listerial effect of bacteriocin or combination treatment in traditional fermented meat sausages	✓ *L. monocytogenes* was undetectable in the samples treated with PA-1 or *P. acidilactici* HA-6112 stored for 14 and 21 days, respectively ✓ Combination treatment was effective in elimination of pathogens indicating immediately or 72 h after HHP, indicating synergistic effects of bacteriocin and HHP	[96]
Synergistic effect of pediocin bacHA-6111-2 (in situ and ex situ) in combination with HHP	Control of *L. innocua* in fermented meat products	✓ The combination treatment effectively controlled *L. innocua* in fermented meat products	[97]
Cooked ham treated with plantaricin BM-1 5120 AU/g, sodium nitrite 0.075 mg/g, and ultra-high-pressure technology (400 MPa for 5 min)	Control of *L. monocytogenes* in cooked ham vacuum packaged and stored under refrigerated conditions for 56 days	✓ Treated samples had reduced *L. monocytogenes* counts (5.79 log CFU/g) at the end of storage ✓ Combination of plantaricin BM-1 with sodium nitrite, ultra-high-pressure technology had a synergistic effect to inhibit *L. monocytogenes* in cooked ham	[98]
Pullulan films containing lauric arginate (LAE) alone or in combination with nisin Z (produced by *L. lactis* subsp. Lactis I8-7-3)	Effect of pullulan film on cooked deliham slices vacuum-packaged and stored at refrigerated temperature up to 28 days	✓ The combination treatment reduced *S. aureus* and *L. monocytogenes* Scott A inoculated onto ham surfaces by approximately 5.53 and 5.62 log_10_ CFU/cm^2^, respectively, during refrigerated storage	[99]
Sakacin-59 (Sak-59) of ***Latilactobacillussakei*** strain	Inhibitory activity against meat spoilage bacteria	✓ Exhibited antimicrobial effects on both Gram-positive (*L. monocytogenes*, *S. aureus*) and Gram-negative (*Serratia marcescens* and *E. coli)* bacteria, but not against the tested Lactobacilli strains	[100]
Bac + strains viz. *Latilactobacillus curvatu*, *L. lactis*, *Pediococcusacidilactici*, *Enterococcus faecium* (Bac + LAB) and Bac + supernatants cell-free (CFS) mixtures	Effect of surface application of Bac + LAB and Bac + CFS mixtures to prevent the growth of *L. monocytogenes* in vacuum-packaged RTE meats (hot dogs- beef and pork trimmings) stored at 5 °C up to 12 weeks	✓ Treated samples had >2-log decrease of *L. monocytogenes* and 6–7 log difference vs. controls during the 12-week challenge study ✓ Cocktail of natural antimicrobial bacteriocins had synergistic effect in inhibiting *L. monocytogenes* in RTE meats	[101]
*L. plantarum* SC01 bacteriocin microencapsulated in 2.5% alginate -6.0% gelatin, *w*/*v* (ALG-GEL) capsules	Antimicrobial activity of bacteriocin and physical quality of pork meat stored in room temperature for 48 h	✓ ALG-GEL formulation of bacteriocin had a maximum inhibitory effect on pathogenic bacteria in fresh pork over a 12 h storage period ✓ Treated pork meat had significantly lower total bacterial count after storage for 12 h and 24 h, compared with the control	[102]
Pediocin bacteriocin from *P. pentosaceus* in combination with *Murraya koenigii* berries (MKB)	Anti-listerial and antimicrobial effects of pediocin on raw goat meat emulsion inoculated with *L. innocua* stored under refrigerated conditions for 9 days	✓ Treated sample has a substantial reduction (*p* < 0.05) in the *L. innocua* count during the entire storage period ✓ Treated sample had significantly (*p* < 0.05) lower aerobic plate count and psychrophilic count ✓ TBARS values found to be lower in MKB-treated samples ✓ Treated and untreated samples had no marked differences for color attributes (L*, a*, b*, hab, C*, ∆E and browning index) ✓ The results for anti-listerial activity of pediocin could be comparable to the nitrite in the raw goat meat emulsion	[103]
Bacteriocin and non-bacteriocin producer strains of *Lactiplantibacillus plantarum*	Enumeration of *L. monocytogenes* of pork colonial sausages	✓ Both the strains equally reduced *L. monocytogenes* count by 1.7 log CFU/g	[33]
Plantaricin BM-1 bacteriocin from *L. plantarum* BM-1	Antimicrobial effect of plantaricin BM-1 on fresh pork chill stored for 7 days	✓ Anti-listerial effect of bacteriocin observed in fresh pork contaminated with *L. monocytogenes* ✓ Plantaricin BM-1 bacteriocin significantly (*p* < 0.01) inhibited the aerobic counts by 1·5 log CFU/g during storage study ✓ Treated pork samples had significantly lower pH, TVBN and higher shelf-life than the control	[104]
Sucrose 0.3% and 1.2% and *L. plantarum*	Effect of combination treatment on chemical, textural and sensory characteristics of Isan sausage stored for 28 days	✓ Sucrose levels and inoculation of *L. plantarum* significantly reduced the TBARS and TVBN values ✓ On sensory evaluation, treated sausages exhibited a higher hardness texture attribute, as well as more intense flavor and a darker color ✓ Ideal combination of sucrose and LAB for production of Isan sausage was 0.3% and 7 log CFU/mL	[105]

HHP: High hydrostatic pressure; LAB: Lactic acid bacteria; MAP: Modified atmosphere packaging, RTE: Ready-to-eat; TBARS: Thiobarbituric acid reactive substances; TVBN: Total volatile basic nitrogen.

## Data Availability

Not applicable.
